# 

**Published:** 1895-08-31

**Authors:** 


**Notices of Births, Deaths, and Marriages** are inserted in this column at a charge of 2s. 6d. for an announcement not exceeding four lines.

**Notice to Advertisers and Subscribers.**

All Advertisements, Orders for Copies of The Hospital and Business Communications should be addressed to **The Manager, NOT TO THE EDITOR,** The Hospital, 428, Strand, London, W.C.

The Cost of Subscription, post free, is as follows:—Per week, 2½d.; per quarter, 2s. 8d.; per half-year, 5s. 3d.; per annum, 10s. 6d. Foreign:—Per Annum, 15s. 2d.; payable, in all cases, in advance.

**NOTICE TO CORRESPONDENTS.**

All MS., Letters, Books for Review, and other matters intended for the Editor should be addressed The Editor, The Lodge, Porchester Square, London, W.

The Editor cannot undertake to return rejected MS., even when accompanied by stamped directed envelope.

**"Burdett's Hospital Annual," 1895.**

Sixth year of publication. 950 pp. Scarlet cloth, gilt lettered. Price 5s. A most complete guide to British, Colonial, Indian, and American Hospitals, Dispensaries, Nursing and Convalescent Institutions, and Asylums. A few copies of the "Annual" for 1894 may still be obtained on application to the Publishers, The Scientific Press (Limited), 428, Strand, London, W.C.

**NOTICE.—Vol. XVII. of "The Hospital" (October, 1894, to April, 1895), in handsome cloth binding, is on sale at the Office of this Journal. Price 6s. Cases for binding the Numbers contained in this Volume, 1s. 6d. Index to Vol. XVII., 2½d., post free.**

**The Monthly Part for September will be ready on September 1st, price 6d.; by post, 8d.**

### SCRAPS AND GLEANINGS.

Bradford Corporation has purchased Brierley Hall for a small-pox hospital.

The Town Council of Eastbourne recently decided to provide covers for all their dust carts.

The late Miss Clara Catherine Warren has left £100 to the Taunton and Somerset Hospital.

The admission of small-pox is not contemplated in connection with the proposed isolation hospital at Crewe.

A report from Ohio speaks of the probable erection and equipment of a Laboratory of Hygiene in connection with the College of New Jersey.

In spite of last year's commercial depression the working-men of Sunderland maintained their subscriptions to the infirmary in a most laudable fashion.

Rathmines has the enviable distinction, according to Dr. Montgomery Ward, of having the lowest death-rate of any town or township in Great Britain or Ireland.

The water supply of Bombay is so great that the surplusage daily brought into the city is stated to lead to waterlogging of the soil, which is a fertile source of disease.

It was decided at the Bordeaux conference that all measures connected with the sanitation of schools must be carried out with the sanction of the Superior Council of the Public Health.

The out-patients at Sunderland Infirmary were increased by 1,560 last year. The total cost of stimulants for in-patients during the same period was £37, or just a little over 3¾d. per head.

At the quarterly court of governors held in August at the Brompton Hospital for Consumption it was announced that the committee had decided on the introduction of electric light.

There is a balance in hand of £567 at the Manchester and Salford Lock Hospital. This charity serves as a hospital, a rescue home, and a hospital for the neighbouring penitent aries.

On the occasion of the annual distribution of prizes to the nurses at Bristol General Hospital, refreshments for the guests were generously provided by Mr. Fry, who took the chair at the meeting.

The Erie County Hospital, U.S.A., contains accommodation for 350 patients, and has six consultants and twenty-two "visiting" doctors. The new wing for consumptive patients has been admirably designed.

The late Mr. William List, J.P., has left £100 each to Cheshunt College, the Orphan Working School, Haverstock Hill, the Royal Hospital for Incurables, Putney, and the Earlswood Asylum for Idiots.

Three separate charges of cattle stealing in Forfarshire were recently brought against a student of St. Andrews University, which resulted in a sentence of three years being passed upon him in the Dundee Justiciary Court.

The Emperor of Germany shot fifty brace of birds on the Wemmergill Moors the other day. His Majesty appears to find no difficulty in manipulating his gun with the right arm only, his left arm being, of course, unavailable.

Dr. Bond, Medical Officer of Health for Southwark, urges the necessity for measles being made a notifiable disease. He states that last year over 3,000 deaths were due to measles, whilst the fatal cases of scarlet fever decreased considerably owing to preventive measures.

The Heatherdene Convalescent Home is a valuable auxiliary to Sunderland Infirmary, and the new wing, which will be completed this autumn, will provide beds for male patients. Hitherto, the accommodation has only sufficed for the women and children sent from the infirmary.

The hospital for infectious diseases which has been erected in the Abassiyeh Desert, near Cairo, is the first building of the kind in Egypt. Hitherto tents have been used for isolating purposes in that country. The new hospital consists of five pavilions, and the disinfecting and laundry departments have been arranged on the most approved modern methods.

The Medical Officer of Health reports of the rivers Holme and Colne at Huddersfield that "they enter the borough in a filthier state than when they leave it. Last year no fewer than 600 bodies of dogs and cats were taken out of the streams and canals in the borough, and no doubt many of them had been washed down from districts lying above Huddersfield."

Mr. George Woofinden has left bequests to the amount of £120,000 to charitable institutions, several of which will receive £2,300 each. The National Lifeboat Institution will get £700. One-third of the residue is for the erection and endowment of almshouses at Sheffield, whilst the remaining two-thirds are intended for a convalescent home, which is to be within twelve miles of the same town.

Mr. and Mrs. Yates Thompson have given £1,000 to the Crewe Memorial Hospital, and have furnished one ward; an equal amount has been contributed by Mr. Webb, chairman of the committee, who has also furnished a ward. The trustees of the late Alderman Heath have handed over £650, and the Hon. R. Ward, M.P., has undertaken to furnish the third ward of this favoured little hospital.

Children are not allowed to work in French factories under the age of thirteen, except in cases where, having obtained the Primary Education Certificate between the age of twelve and thirteen, they also satisfy the doctor as to their physical fitness. The medical examination is compulsory before thirteen, and optional from that to sixteen.

In addition to some unusually fine art needlework, and other contributions of rare beauty, the bazaar lately held in aid of the Chalmer's Hospital was distinguished by the presence of some original pictures by Mr. Arthur Fraser, Mr. A. T. Purnell, Mr. Churcher, Mr. de Lacy, Mr. T. C. Hook, R.A., Mr. Colin Hunter, R.S.A., Mr. Dudley Hardy, and Mr. Mark Anderson (Cynicus), which were sold for the charity.

The first annual report of the Royal Victoria Hospital, Montreal, is rendered attractive by good type and excellent illustrations, through which the reader is made familiar with the appearance and administration of this handsome hospital. It contains three theatres devoted respectively to operations, medical teaching, and post-mortems. Each is most complete in its way, and admirably adapted for the purpose for which it has been designed.

### THE STUDENT OF MEDICINE.

### APPOINTMENTS.

W. M. Clendinnen, M.R.C.S.Eng., L.R.C.P.Lond., appointed Medical Officer of Sedgley No. 3 District of the Dudley Union. H. C. Heathcote, M.B., Ch.B., appointed Medical Officer for the Fifth District of the Bath Union. Dr. L. F. Houghton, appointed Medical Officer for the Second District of the Liskeard Union. Dr. Jenkins, reappointed Medical Officer of Health for Lytham. E. C. Kingdon, M.B., M.R.C.S., appointed Honorary Surgeon to the Nottingham Eye Infirmary. A. A. Mackeith, M.B., C.M.Glasg., appointed Medical Officer for the Seventh District of the Ba-nstaple Union. Ahmed Mirza, M.B., B.Sc. Edin., Medical Officer for the City of Hyderabad, appointed Lecturer on Medical Jurisprudence at the Medical School at Hyderabad. Dr. R. W. Nairn, appointed Medical Officer for the Farnsfield District of the Southwell Union. Mr. W. G. Nash, appointed Medical Officer for the No. 7 District of the Market Harborough Union. W. S. Nason, M.B., C.M.Edin., appointed Medical Officer for the Nuneaton Union Workhouse. Michael O'Sullivan, M.B., B.Ch., appointed Medical Officer for the C, D, and G Divisions of the Dublin Police. H. C. Perkins, M.R.C.S., L.R.C.P., appointed Medical Officer Nayar Brigade, Travancore, Madras. M. B. Ray, M.B., C.M.Edin., appointed Resident Medical Officer to Wadsley Asylum, near Sheffield. R. W. Innes Smith, M.B., appointed Assistant House Surgeon at Ancoats Infirmary, Manchester. C. Templeman, M.D., D.Sc., appointed Medical Officer of Health for the City of Dundee. G. J. K. Turner, M.B., C.M.Aberd., appointed Medical Officer for the No. 4 District of the Chipping Norton Union. Dr. F. H. R. J. A. Walker, appointed Medical Officer for the Coleford District of the Frome Union. J. A. Henton White, M.B., B.S. Dunelm, L.R.C.P., M.R.C.S.Eng., appointed Resident Surgical Officer to the General Dispensary, Birmingham.

ST. THOMAS'S HOSPITAL.—The following gentlemen have been selected as house officers from Tuesday, September 3rd:—Resident House Physicians: E. G. C. Daniel, B.A.Cantab., L.R.C.P., M.R.C.S. (extension); L. L. Jenner, M.A., M.B., B.Ch Oxon., M.R.C.P. (extension). Non-Resident House Physicans: P. J. A. Seccombe, M.A.Cantab., L.R.C.P., M.R.C.S.; F. G. Layton, L.R.C.P., M.R.C.S. House Surgeons: E. O. Thurston, L.R.C.P., M.R.C.S.; A. L. Home, L.R.C.P., M.R.C.S.; W. G. Stone, M.A., M.B., B.Ch.Oxon., L.R.C.P., M.R.C.S.; and H. J. Davis, M.A., M.B., B.C.Cantab., L R.C.P., M.R C.S. Assistant House Surgeons: L. A. R. Wallace, B.A., M.B, B.Ch.Oxon., L.R.C.P., M.R.C.S.; H. C. Crouch, L.R.C.P., M.R.C.S.; J. L. Prain, L.R.C.P., M.R.C.S.; and G. J. Conford, B.A., M.B., B.Ch.Oxon., L.R.C.P., M.R.C.S. Obstetric House Physicians: Senior, G. Candler, B.A.Cantab., L.R.C.P., M.R.C.S.; Junior, E. A. Saunders, M.A., M.B., B.Ch.Oxon., L.R.C.P., M.R.C.S. Clinical Assistant in the Special Department for Diseases of the Throat: J. W. Cornwall, M.A., M.B., B.C.Cantab. Clinical Assistants in the Special Department for Diseases of the Skin: G. G. Genge, L.R.C.P., M.R.C.S., and G. B. C. Blount, L.R.C.P. M.R.C.S. Clinical Assistants in the Special Department for Diseases of the Ear: H. H. Haward, B.A.Cantab., L.R.C.P., M.R.C.S., and E. W. Palin, M.A., M.B., B.Ch.Oxon., L.R.C.P., M.R C.S. Clinical Assistants in the Electrical Department: W. E. Dixon, B.Sc.Lond., L.R.C.P., M.R.C.S. (extension); Ophthalmic House Surgeon (Junior): A. H. P. Dawnay, L R.C.P., M.R.C.S.

### VACANCIES.

The following vacancies are announced this week, the amount of salary in each case being given after the name of the institution:—

#### Resident Medical Officers.

Brighton, Hove, and Preston Dispensary, Queen's Road, Brighton.—Medical Officer for the No. 6 District. Applications to the Secretary by September 9th.

City of London Hospital for Diseases of the Chest, Victoria Park, E.—House Physician. Board and residence and allowance for washing provided. Appointment for six months. Applications to the Secretary by September 12th.

General Hospital, Nottingham.—House Physician. Appointment for two years, but eligible for re-election. Salary £100 per annum, rising £10 a year to £120. Assistant House Surgeon. Appointment for six months. Board, lodging, and washing in hospital; no salary. Applications to the Secretary for the former post by September 11th, and for the latter by September 7th.

Royal Free Hospital.—Senior Medical Officer, doubly qualified. Salary £100 per annum, with board, residence, and washing. Applications to Conrad W. Thies, Secretary, by September 28th.

### THE STUDENT OF MEDICINE—*continued.*

Manchester Clinical Hospital for Women and Children, Park Place, Cheetham Hill Road, Manchester.—House Surgeon. Salary £80 per annum, with apartments and board. Applications to Mr. Hubert Teague, Secretary, 38, Barton Arcade, Manchester, by September 3rd.

Norfolk and Norwich Hospital.—Assistant House Surgeon, doubly qualified. Appointment for six months. Board, lodging, and washing provided. Applications to the House Surgeon by September 2nd.

Royal Southern Hospital, Liverpool.—Third House Surgeon. Salary £65 per annum, with board, lodging, and laundry. Applications to the Chairman of the Medical Board by August 26th.

West Ham Hospital, Stratford, E.—Senior House Surgeon. Appointment tenable for one year. Salary £75 per annum, with board, lodging, and washing. Applications to G. E. Adams, Secretary, by August 31st.

West Riding Asylum.—Fifth Assistant Medical Officer. Salary £100 per annum, rising £10 a year up to £150, with board, &c. Applications to the Medical Superintendent by August 28th.

#### Non-Resident Medical Officers.

City of London Hospital for Diseases of the Chest, Victoria Park, E.—Assistant Physician; must be M. or F.R.C.P.Lond. Applications to the Secretary by September 14th.

Glasgow Maternity Hospital.—Obstetric Physician and Assistant Obstetric Physician. Applications to Arthur Forbes, Secretary, 146, Buchanan Street, Glasgow, by November 8th.

School Board for London.—Medical Officer for the Board's Training Ship *Shaftesbury*, lying off Grays, Essex; must reside within two miles of the ship. Commencing salary £100 a year, which may be increased by annual increments of £10 to £150 a year. Applications to A. E. Garland, Clerk to the Managers, School Board Offices, Victoria Embankment, W.C., by August 31st.

### UNIVERSITIES AND COLLEGES.

#### University of London.

Intermediate Examination in Medicine.—Pass List:

Entire Examination.—First Division: W. R. Battye, B.Sc. University Colleges, Bristol and London; Janet Waldegrave Carr, London School of Medicine for Women; H. Hartley, Owens College, Manchester; Alice Mary Hawker, London School of Medicine for Women; H. Hine, Middlesex Hospital; H. D. Singer, St. Thomas's Hospital; F. H. Thiele, University College; W. B. H. Wood, Mason College. Second Division: E. W. Adams, Sheffield Medical School and Firth College; F. A. Arnold, London Hospital; C. H. Benham, University College; T. P. Berry, Guy's Hospital; F. Brickwell, St. Bartholomew's Hospital; J. Broadley, Yorkshire College; S. A. Bull, Westminster Hospital; A. J. Cleveland, Guy's Hospital; R. M. Cowie, King's College; D. L. F. Davies, Middlesex Hospital; R. C. Field, Yorkshire College; G. M. Harston, Charing Cross Hospital; T. A. Hawkesworth, King's College; W. J. Hirst, Yorkshire College; H. H. Jamieson, St. George's Hospital; H. S. Jenkins, University College, Bristol; J. D. Jenkins, London Hospital; R. C. Leaning, St. Mary's Hospital and Birkbeck Institute; Frances Annie Leete, London School of Medicine and Birkbeck Institute; C. D. Lindsey, St. Mary's Hospital and Birkbeck Institute; Marion Sandford Linton, B.A., London School of Medicine for Women; F. S. Lloyd, St. Mary's Hospital; J. Mooney, Owens College; J. B. Page, St. Mary's Hospital; Mary Nona Sharman, Glasgow University and London School of Medicine; J. H. Sheldon, Owens College; A. B. Smallman, Owens College; J. E. Smith, University College, Liverpool; S. M. Smith, St. Mary's Hospital; J. M. G. Swainson, Westminster Hospital; E. Taunton, University College; W. H. M. Telling, Guy's Hospital; A. G. Tollputt, University College, Dundee, and Queen's, Belfast; W. F. Tyndale, St. George's Hospital; Eliza Turner Watts, London School of Medicine for Women; H. E. White, Mason College; C. B. Whitehead, St. Mary's Hospital; W. D. Wiggins, St. Mary's Hospital; P. G. S. Williams, University College; A. G. Wilson, London Hospital; W. Wright, Owens College.

Excluding Physiology.—First Division: W. T. Rowe, St. Bartholomew's Hospital; H. H. Scott, St. Thomas's Hospital; G. B. Thwaites, St. Thomas's Hospital; J. G. Wallis, London Hospital. Second Division: H. T. S. Aveline, Bristol Medical School and Clifton Laboratory; F. V. O. Beit, St. Bartholomew's Hospital; A. B. Cridland, Bristol Medical School and Clifton Laboratory; G. B. Crisp, St. Mary's Hospital; L. E. C. Handson, Guy's Hospital; J. L. Jones, University College; J. A. Mawson, Yorkshire College; H. A. Schölberg, St. Bartholomew's Hospital; S. R. Smith, Westminster Hospital; W. S. V. Stock, University College, Bristol, and Clifton Laboratory; L. Whitfield, Mason College; E. W. Woodbridge, St. Bartholomew's Hospital.

Physiology Only.—First Division: A. E. Baker, Middlesex Hospital. Second Division: J. A. P. Barnes, St. Bartholomew's Hospital; G. P. Bletchly, Middlesex Hospital; W. N. East, Guy's Hospital; E. S. Hall, Guy's Hospital; E. L. Hunt, St. George's Hospital; C. E. Lansdown, St. Mary's Hospital; H. J. Marriage, St. Thomas's Hospital; J. L. Maxwell, St. Bartholomew's Hospital; P. W. Moore, Guy's Hospital; J. W. Winterburn, St. Thomas's Hospital.

Intermediate Examination in Medicine for Honours.

Anatomy.—First Class: *C. H. Fagge, Guy's Hospital; and †F. E. Walker, Guy's Hospital. Second Class: W. A. Dewhirst, Yorkshire College, and E. B. Sherlock, B.Sc., Westminster Hospital (equal). Third Class: E. C. Morland, B.Sc., Owens College and St. Bartholomew's Hospital; M. Dixon, B.Sc., University College; and H. E. Hewitt, St. Thomas's Hospital.

Physiology and Histology.—First Class: *E. C. Morland, Owens College and St. Bartholomew's Hospital; †R. Kelsall, Owens College; and F. E. Walker, Guy's Hospital. Second Class: J. F. Dobson, Yorkshire College, and C. Dykes, University College (equal); E. B. Sherlock, Westminster Hospital; and G. E. Richmond, B.A., B.Sc., Guy's Hospital and King's College. Third Class: Mary Frances Cornford, London School of Medicine and University College, and P. Turner, B.Sc., Guy's Hospital (equal).

Organic Chemistry.—First Class: *R. W. C. Pierce, B.Sc., St. Thomas's Hospital. Second Class: P. Turner, Guy's Hospital; W. T. Milton, Guy's Hospital; E. C. Morland, Owens College and St. Bartholomew's Hospital; E. B. Sherlock, Westminster Hospital; and G. E. Richmond, Guy's Hospital and King's College. Third Class: H. W. Bruce, Guy's Hospital; and D. N. Nabarro, B.Sc., University College.

Materia Medica and Pharmaceutical Chemistry.—First Class: *P. Turner, Guy's Hospital; ‡E. B. Sherlock, Westminster Hospital; W. T. Milton, Guy's Hospital; and F. E. Walker, Guy's Hospital. Second Class: R. W. C. Pierce, St. Thomas's Hospital; T. H. Gardner, King's College; H. E. Hewitt, St. Thomas's Hospital. Third Class: H. W. Bruce, Guy's Hospital; Mary Frances Cornfield, London School of Medicine and University College; and C. H. Fagge, Guy's Hospital.

* Exhibition and Gold Medal. † Gold Medal.

‡ Obtained the number of marks qualifying for the Exhibition.

